# “I left my country and the people I love. It gave me hypertension”: a qualitative study of social support and hypertension management in refugees

**DOI:** 10.3389/fpubh.2026.1849878

**Published:** 2026-07-15

**Authors:** Ariana Khayamian, Carine Tamamian, Lana Bridi, Alissa B. Sideman, Nour Makarem, Dahlia A. Kaki, Tala Al-Rousan

**Affiliations:** 1 Displacement and Health Research Center, Herbert Wertheim School of Public Health and Human Longevity Science, University of California, San Diego, La Jolla, CA, United States; 2 School of Medicine, University of California, San Diego, La Jolla, CA, United States; 3 Philip R. Lee Institute for Health Policy Studies, University of California, San Francisco, San Francisco, CA, United States; 4 Global Brain Health Institute, University of California, San Francisco, San Francisco, CA, United States; 5 Department of Epidemiology, Mailman School of Public Health, Columbia University Irving Medical Center, New York, NY, United States; 6 Department of Emergency Medicine, Los Angeles General Medical Center, Los Angeles, CA, United States

**Keywords:** health outcomes, hypertension management, medication adherence, refugee health, social connectedness, social support theory

## Abstract

**Background:**

Refugees experience a disproportionately high burden of hypertension, with war-related displacement disrupting their social networks. Countries from the Middle East and North Africa (MENA) are among the largest producers of refugees globally. While previous research has established the role of social support in improving chronic disease management, little is known about its impact among refugees from Arabic-speaking MENA countries. This study focuses on refugees diagnosed with hypertension from two Arabic-speaking MENA countries, Syria and Iraq, to investigate perceived social connectedness and the influence of social support on hypertension management.

**Methods:**

107 refugees from Iraq (*n* = 84) and Syria (*n* = 23) who resettled in San Diego, CA, were recruited from a federally qualified health center in a refugee-majority neighborhood. Participants completed quantitative surveys; a subsample of 54 completed in-depth interviews until thematic saturation was reached.

**Results:**

Mean age was 61.61 years (SD = 9.81), mean years since United States resettlement was 9.6 years (SD = 5.82), and 48.6% (*n* = 52) of participants were female. Overall, 60.7% (*n* = 65) reported social disconnection, and 61.7% reported an annual income <$15,000. Participants described 3 forms of social support (or lack thereof) that impact hypertension management. (1) Informational: reliance on close social networks for health information; (2) Instrumental: lack of transportation and language barriers in clinical spaces; and (3) Emotional: loss of social networks and social status, causing psychosocial stress.

**Conclusion:**

Findings suggest that prioritizing family reunification during resettlement and integrating social support into public health interventions, such as family-centered care, shared medical appointments, and community-based peer support, may mitigate the loss of social support and related hypertension disparities among refugees.

## Introduction

Hypertension is a leading global cause of preventable illness and death, responsible for approximately 11 million deaths annually, representing 16% of all deaths worldwide, and is attributable to more than half of all cardiovascular mortality globally (1–4). In the United States (US), hypertension imposes an estimated economic burden of $131 billion per year (5). Hypertension incidence increases with age, affecting over 76% of adults over 65 (6), and control rates remain low among minoritized communities, where structural inequities continue to drive health disparities (7). These trends highlight the necessity of early hypertension management by ensuring proper and timely access to healthcare.

Simultaneously, the global refugee population continues to grow, with one in every 78 individuals forcibly displaced. Over the past decade, the US has admitted nearly 470,000 refugees, with Syrians and Iraqis comprising two of the largest groups at 11% and 7.4% of total admissions, respectively (8). Refugees face unique health challenges, including chronic stress, inequitable health insurance access, linguistic and cultural barriers, and disruptions to care (9–11). Among immigrant populations, refugees have demonstrated a greater prevalence of hypertension, with rates between 12 and 14%, compared to 6 to 9% among non-refugee immigrant adults (12). A prevalence study in a US refugee clinic also found that more than a third of refugees were diagnosed with hypertension, with uncontrolled hypertension rates as high as 46.7% (13). As a result, hypertension management remains a persistent barrier within this population, which remains one of the most understudied groups in hypertension research (13).

Refugees from Syria and Iraq, part of the Arabic-speaking Middle East and North Africa (MENA) region, face added challenges to hypertension management, including financial hardship and limited health literacy (11). Prior to displacement, approximately 25% of the adult Syrian population and 33% of Iraqi refugees screened before US resettlement had hypertension, with an additional 42% of Iraqis diagnosed as pre-hypertensive (14, 15). Recent studies among Syrian and Iraqi refugees in the US have reported hypertension prevalence rates of 61%, which is substantially higher than most other immigrant groups (16). Existing evidence suggests that social support can reduce physiological responses to stress, which are established risk factors for hypertension (12). However, literature is scarce on effective hypertension management interventions that align with the distinct cultural values of refugees. Culturally tailored interventions, which are sensitive and effective for chronic disease self-management among minority groups, can improve health outcomes and patient satisfaction (17, 18). Further investigation is needed to identify the role and significance of cultural factors in healthcare most relevant to refugee populations.

Social support has been associated with better hypertension control through improved health literacy and medication adherence in disadvantaged populations (19–22). This is particularly pertinent for refugee populations, who frequently encounter challenges in health proficiency and literacy (23). Conversely, social isolation, common among refugees, is linked to higher rates of chronic disease, including hypertension (24, 25). Residing in neighborhoods with low social capital compounds this risk, as these residents tend to perceive their health status as poor compared to those with greater social capital (26). For aging refugees, family separation and loss of community amplify this vulnerability (27–29). One of the most profound consequences of displacement is the loss of social support systems, including the loss of home and community ties. Despite growing evidence of high cardiovascular risk and hypertension prevalence among refugees and other migrant populations, there is scant literature on the role of social support in chronic disease management among Arabic-speaking MENA refugees, and almost nothing is known for this specific refugee population (13).

Syrian and Iraqi refugees present a distinct social support context. Arab culture is strongly collectivist, with social support embedded in family and community systems that are disrupted by forced displacement and resettlement (30). Syrian and Iraqi refugees have experienced compounded losses, including war trauma, prolonged displacement, family separation, and loss of social status, which may disrupt social support systems in ways that are not well captured by existing literature (27, 28, 31). Structural barriers to hypertension management, specific to this population, including Arabic language isolation, restrictive immigration policies affecting family reunification, and limited culturally concordant resources in resettlement communities, may shape how social support is experienced and accessed (11, 13). These factors suggest that findings from studies of other migrant or refugee groups cannot be assumed to apply to Syrian and Iraqi refugees.

Although the global refugee population is racially and ethnically heterogeneous, a significant proportion of refugees that have resettled in the US over the past two decades have originated from Arabic-speaking MENA countries, with Arab individuals forming one of the largest groups (8). California hosts the largest Arab American population nationwide, with San Diego County positioned as a primary destination for refugee resettlement, particularly among individuals from Iraq and Syria (32, 33). Although the broader region of the MENA encompasses many countries, this study specifically focuses on refugees from Syria and Iraq, who represent one of the largest refugee groups from the Arabic-speaking MENA region globally. Over the past decade, Syria and Iraq have accounted for approximately 18.4% of all US refugee admissions combined (8). Middle Eastern, North African, and Arab cultures emphasize collectivism and interdependence (30), suggesting that social support may serve as a critical component in managing chronic conditions, including hypertension (34). Understanding the role of social support in managing chronic conditions is essential for preparing health and social service systems to meet the needs of aging refugees. To address this gap, this study employs a qualitative approach to examine how social support influences hypertension management among Syrian and Iraqi refugees in San Diego. Specifically, this study addresses the following research questions: (1) What is the prevalence of social connectedness among Syrian and Iraqi refugees with hypertension resettled in San Diego, CA? (2) How does social support, informational, instrumental, and emotional, influence hypertension management in this population?

## Materials and methods

### Design, setting, and subjects

This focused study assessed hypertension self-management among Arabic-speaking refugees in San Diego, California, a major hub for refugee resettlement in the US, for resettling refugees from Arabic-speaking MENA countries fleeing from persecution, conflict, and violence. Participants were recruited between April 2021 and April 2022 through the Family Health Center of San Diego (FHCSD), a federally qualified health center and a major healthcare provider for refugees in San Diego. Inclusion criteria were: (1) diagnosis of hypertension (confirmed through FHCSD electronic health records); (2) present or former refugee status; (3) Iraqi or Syrian origin; and (4) residence in San Diego. Individuals under 21 years old or unable to provide informed consent were excluded. From a FHCSD list of patients with hypertension, potential participants were randomly contacted via phone. Of 756 calls, 17.20% of individuals met the eligibility criteria (130 of 756); of those eligible, 20 declined participation, and 110 were enrolled. Oral informed consent was obtained from each participant and documented by a trained research assistant who signed on the participant's behalf. The participant flow is summarized in the STROBE flow diagram (Supplementary Figure 1). Following screenings and consent, participants completed an in-person interview at the Majdal Community Center, a community-based organization and community partner in El Cajon, a city within San Diego County.

### Data collection and measures

Quantitative data were collected via electronic surveys, administered either in person or virtually on Zoom, based on reported participant preference and in accordance with local COVID-19 restrictions. Interviewers read questions aloud to participants and recorded their responses. Participant characteristics included: gender (collected via FHCSD electronic health records), age, country of origin, date of US resettlement, employment status, annual household income, marital status, ability to read and write in Arabic and English, highest level of education attained, and emergency contacts. Qualitative data were collected following survey completion through 50-min in-depth interviews. 54 interviews were conducted using a semi-structured interview guide (Supplementary Appendix 1), which covered domains including demographics, social connectedness and support, and barriers to hypertension management. All 107 participants completed the quantitative survey. In-depth interviews were subsequently conducted with consecutively enrolled, consenting participants until thematic saturation was reached; no new themes emerged after 54 interviews, at which point data collection ceased. With participant consent, interview audio was recorded, transcribed, and translated from Arabic to English.

The primary outcome variable, social connectedness, was measured using a single survey question assessing perceived social connectedness: “*Do you consider yourself socially connected to a specific community or cultural group in San Diego?*” Response options were: “*Yes*,” “*No*,” and “*I don't know*.” Responses of “I *don't know*” were excluded from final data analysis, yielding a binary Yes/No outcome variable. Sociodemographic characteristics were collected for descriptive purposes and selected based on available data from prior literature, including age, gender, marital status, annual household income, education level, employment status, country of origin, Arabic literacy, English literacy, household size, and number of children. Marital status was re-coded into two categories: currently married and not currently married. Annual income and education level were organized into three and four categories, respectively, to account for the low number of observations. Emotional support questions were adapted from the Oslo Social Support Scale (35) and included: number of close friends and number of emergency contacts.

### Analysis and theoretical framework

Quantitative data were analyzed using Statistical Package for the Social Sciences version 28.0 (SPSS v28.0) to generate descriptive statistics characterizing the sociodemographic profile of the study sample. Frequency distributions and proportions were used to summarize participant characteristics. Descriptive quantitative findings (Table 1) aim to contextualize the qualitative sample and support the interpretation of qualitative findings. The Strengthening the Reporting of Observational Studies in Epidemiology (STROBE) guidelines were used for reporting the descriptive quantitative results (36).

**Table 1:** Socio-demographic characteristics of the participant Syrian and Iraqi refugee population in San Diego by social connectedness.

Socio-demographic characteristics	Social connectedness *n (%)* 42 (39.3)	No social connectedness *n (%)*65 (60.7)	Total *n (%)* 107
Age (mean, SD)	60.21 (11.03)	62.23 (8.93)	61.61 (9.81)
Gender			
Male	24 (57.1)	31 (47.7)	55 (51.4)
Female	18 (42.9)	34 (52.3)	52 (48.6)
Country of origin			
Iraq	30 (71.4)	54 (83.1)	84 (78.5)
Syria	12 (28.6)	11 (16.9)	23 (21.5)
Marital status			
Currently married	35 (83.3)	54 (83.1)	89 (83.2)
Not currently married	7 (16.7)	11 (16.9)	18 (16.8)
Educational attainment			
Less than high school	16 (38.1)	27 (41.5)	43 (40.2)
High school	11 (26.2)	15 (23.1)	26 (24.3)
Vocational certificate	4 (9.5)	11 (16.9)	15 (14.0)
Bachelor's degree or higher	11 (26.2)	12 (18.5)	23 (21.5)
Annual household income			
Less than $15,000	30 (71.4)	36 (55.4)	66 (61.7)
$15.001—$25,000	6 (14.3)	25 (38.5)	31 (29.0)
More than $25,000	6 (14.3)	4 (6.2)	10 (9.3)
Employment status			
Employed	6 (14.3)	8 (12.3)	14 (13.1)
Unemployed	36 (85.7)	57 (87.7)	93 (86.9)
**Arabic literacy**			
Yes	39 (92.9)	57 (87.7)	96 (89.7)
No	3 (7.1)	8 (12.3)	11 (10.3)
English literacy			
Yes	18 (42.9)	18 (27.7)	36 (33.6)
No	24 (57.1)	47 (72.3)	71 (66.4)
**Household size**			
1–2	14 (34.1)	21 (32.3)	35 (33.0)
3–4	11 (26.8)	24 (36.9)	35 (33.0)
5 or more	16 (39.0)	20 (30.8)	36 (34.0)
Number of children			
4 or less	27 (67.5)	39 (60.9)	66 (63.5)
5 or more	13 (32.5)	25 (39.1)	38 (36.5)
Number of emergency contacts			
1	8 (19.0)	13 (20.0)	21 (19.6)
2	10 (23.8)	14 (21.5)	24 (22.4)
3	9 (21.4)	14 (21.5)	23 (21.5)
4	6 (14.3)	11 (16.9)	17 (15.9)
5 or more	9 (21.4)	13 (20.0)	22 (20.6)
Reported number of close friends within community			
1	6 (14.3)	18 (27.7)	24 (22.4)
2	5 (11.9)	7 (10.8)	12 (11.2)
3	9 (21.4)	11 (16.9)	20 (18.7)
4	5 (11.9)	8 (12.3)	13 (12.1)
5 or more	17 (40.5)	21 (32.3)	38 (35.5)

3 participants (2.8%) responded “I don't know” to the social connectedness question and were excluded from analysis, resulting in a total of 107 participants with valid responses. Percentages may not sum to 100% due to rounding. Percentages are calculated with *n* = 107 as the denominator (*n* = 42 social connectedness; *n* = 65 no social connectedness) except where noted. Missing values accounted for differences in denominators for the Number of Children variable (3 participants with missing data, *n* = 104 total; *n* = 40 social connectedness; *n* = 64 no social connectedness) and the Household Size variable (1 participant missing in total, *n* = 106; and 1 participant missing in social connectedness, *n* = 41).

Quantitative analyses were conducted to characterize patterns of social connectedness and identify associated sociodemographic factors, while qualitative analyses explored how social support shaped hypertension management experiences.

**Figure 1:**
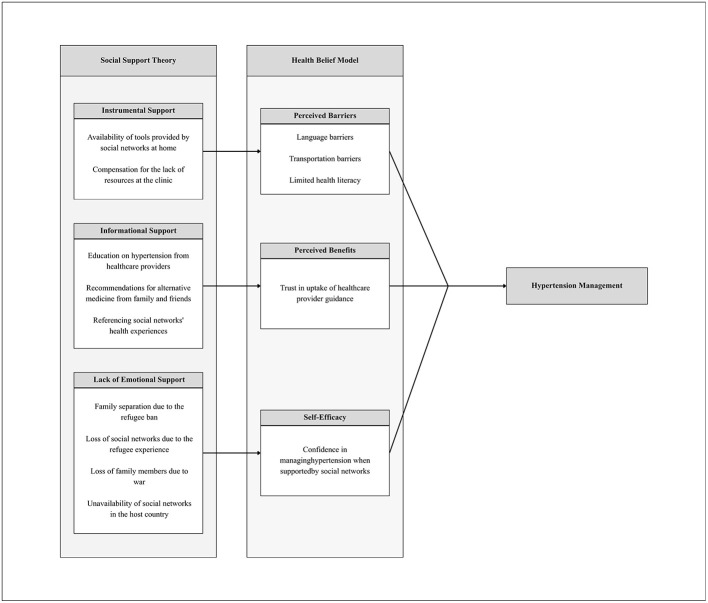
A conceptual framework for the role and availability of social support for Syrian and Iraqi refugees with hypertension. The framework illustrates the correspondence between Social Support Theory domains (Instrumental Support, Informational Support, and Lack of Emotional Support) and Health Belief Model constructs (Perceived Barriers, Perceived Benefits, and Self-Efficacy) in shaping hypertension management. Appraisal support, the fourth domain of Social Support Theory, did not emerge in the qualitative data and is not shown.

Qualitative data were analyzed using inductive thematic analysis using ATLAS.ti and Crabtree and Miller's 5-step interview framework to identify recurring themes in the data (37). Researchers independently coded transcripts, refined the codebook through consensus, and continued until thematic saturation. The study team included bilingual researchers and second-generation refugees with lived experience of forced migration, which facilitated cultural concordance and trust with participants; potential interpretive influences of researcher positionality were mitigated through independent coding and consensus-based codebook refinement. Qualitative results were reported using the Standards for Reporting Qualitative Research (SRQR) (38). Two theoretical frameworks informed this study. Social support theory, which defines four distinct tenets of social support that influence health and wellbeing: informational, instrumental, emotional, and appraisal (39–42), guided interpretation of qualitative themes. Three of the four domains—(1) informational, (2) instrumental, and (3) emotional—directly corresponded to and emerged in the data. These tenets encompassed (1) tangible aid; (2) provision of care, love, and trust; and (3) guidance, advice, and problem-solving (42–44). The Health Belief Model was additionally used to interpret how participants' beliefs and perceived social connectedness influenced their health-related behaviors, such as hypertension management (45, 46). Specifically: (1) perceived barriers corresponded to language barriers, transportation barriers, and limited health literacy documented under Instrumental Support; (2) perceived benefits corresponded to participants' trust in uptake of healthcare provider guidance documented under Informational Support; and (3) self-efficacy corresponded to participants' confidence in managing hypertension when supported by their social networks, documented under Emotional Support. The applied framework (Figure 1) integrates these theoretical constructs, social support theory and the Health Belief Model, to illustrate the role of social support in hypertension management.

### Ethics statement

This study was approved by the Institutional Review Board at the University of California, San Diego. Oral informed consent was obtained from all individual participants prior to enrollment and was documented by a trained research assistant who signed on the participant's behalf, consistent with the University of California, San Diego's Institutional Review Board-approved procedures. The authors affirm that research participants provided informed consent for publication of their quotations and data.

## Results

Table 1 depicts descriptive statistics by social connectedness. Among 107 participants (51.4% male; mean age 61.61, SD = 9.81), most were Iraqi (78.5%) and unemployed (86.9%), with an average US residence of 9.6 years (SD = 5.82). Overall, 39.3% reported social connectedness, while 60.7% did not. Among those with lower annual household income (< $15,000), a higher proportion reported social connectedness (71.4%) compared to those earning more than $25,000 (14.3%).

Participants lacking social connectedness were mostly female (52.3%), Iraqi (83.1%), married (83.1%), with limited English literacy (72.3%), lower educational attainment (41.5% less than high school), and a mean age of 62.2 years (SD = 8.93). Household sizes varied (1–2: 32.3%; 3–4: 36.9%; ≥5: 30.8%). Among those not socially connected, the most common number of emergency contacts was 2–3 (21.5%), while 32.3% reported having ≥5 friends. Question 10 (Supplementary Appendix 1) asked whether participants felt connected to a specific community or group in San Diego, while Question 11 asked how many close friends they had within their community. These questions measure distinct constructs, as a participant can have many close friends and still feel disconnected from their broader community. This is particularly relevant among collectivist cultures, where belonging is defined by community-level integration rather than individual friendships.

Qualitative results highlighted three main themes: (1) trust in informational support from healthcare providers (HCPs) and family; (2) reliance on instrumental support (e.g., transportation, interpretation); and (3) lack of emotional support, which negatively impacted hypertension management.

### Theme 1: informational support as a facilitator

Participants discussed seeking guidance from HCPs and personal networks as essential to effective hypertension management.

### Theme 1.1: informational support in the clinical space

HCPs were trusted sources of guidance for lifestyle modifications, including exercise, diet, and blood pressure monitoring. HCPs also helped participants overcome barriers, like low health literacy, by providing education on hypertension and its complications. Participants emphasized that patient-centered, culturally responsive care enhanced compliance and confidence in treatment.

“*My doctor, who prescribed me my hypertension medication, told me to eat healthily and exercise, so I listen to them.”* [Participant 12]

“*Hypertension worries me because the doctor says if high blood pressure persists, it will cause stroke and health problems.”* [Participant 151]

“*My doctor taught me how to use the blood pressure monitor and write down the readings, and I am happy with their orders.”* [Participant 55]

Participants believed that thorough explanations from HCPs improved adherence:

“*When a doctor and a specialist thoroughly explain the mechanisms and side effects, I would take the medication with no hesitation, but when they don't, I don't care.”* [Participant 54]

Conversely, lack of informational support from HCPs reduced motivation and proactive engagement in hypertension management:

“*If my doctor tells me to measure my blood pressure, I would do it; however, [if] my doctor does not tell me, and I feel normal, [then I would not measure it]. If I ever feel dizzy or have any other symptoms, I [would then] measure my blood pressure*.” [Participant 184]

Trust in HCPs and medical recommendations varied. Some expressed strong confidence, while others insisted on self-research or traditional remedies:

“*[I trust my doctor's advice], when I am around her, I feel so comfortable*.” [Participant 15]

“*My trust in doctors is little. I depend more on herbal medicine and my diet. Since medications are all chemicals and have side effects.”* [Participant 150]

“*I have no trust in American doctors. They just capitalize on my illness.”* [Participant 99]

Shared language and culture with HCPs strengthened trust and communication:

“*I have a Lebanese doctor […] in the US, and he is the best. But when he is not on call, I feel lost with other doctors.”* [Participant 99]

“*Yes, [my relationship with my non-Arab doctor is good], but not like my previous Arab doctor, […]. [My previous Arab doctor] was amazing.”* [Participant 194]

Participants valued clinical encounters that emphasized shared decision-making, honored patient autonomy, and acknowledged cultural values. Positive experiences occurred when HCPs listened attentively and incorporated patient perspectives to tailor support to individual needs:

“*When I see that my blood pressure is high, this triggers my anxiety, and then this anxiety causes my blood pressure to increase. So, when I feel like there is so much pressure going on, I take my medication. I even asked my doctor if I can take two pills of the hypertension medication spaced out during the day, just to make sure I am on the safe side of managing my hypertension, and she said I can.“* [Participant 5]

### Theme 1.2: informational support through close networks

Family, friends, and online communities provided hypertension-related advice, predominantly focused on dietary changes and home remedies. These informal supports complemented clinical care to shape health behaviors.

“*My mother-in-law drinks garlic [for her hypertension], and she recommended it to me. I swallow garlic with water, which is a typical Arab remedy.”* [Participant 4]

“*[My husband] give[s] me my hypertension medications. If we saw that medications are not solving my migraines, he advises me to drink lemon.”* [Participant 106]

“*My friends and other social media accounts [recommended I consume ginger for my hypertension management]. I heard that ginger helps in weight loss and preventing heart attacks.”* [Participant 54]

Participants also sought guidance from medical professionals within their social networks, often receiving advice on lifestyle modifications and medication regimens:

“*My wife's cousin was a doctor… He advised me at that time to lower my salt intake.”* [Participant 143]

“*I reached out to my nephew in Canada, who is a nurse practitioner. He told me to advise my American doctors to prescribe me diuretics and antiarrhythmic medications. Right now, I am using these medications, and I feel better while also avoiding high salt intake and upsetting myself.”* [Participant 5]

Loved ones' experiences acted as a point of reference for health management, shaping participant perceptions and attitudes toward their own hypertension:

“*[I am worried about hypertension] because my mother had hypertension, and it caused her to have a heart attack, and [she] was paralyzed in her arm and leg.”* [Participant 5]

“*I noticed how my friends and relatives here in the US, who also happen to be refugees, have had better health outcomes after following their doctors' orders and taking care of themselves. Some even underwent surgeries and have fully recovered.”* [Participant 71]

### Theme 2: instrumental support

Participants relied on social networks for practical help in managing hypertension, including medication reminders, use of medical devices, transportation, and translation. This tangible assistance was crucial to treatment adherence and accessing care:

“*…My daughter is the one that helps me [use my blood pressure monitor] and measures it for me. I do not know how to use the device.”* [Participant 233]

“*My daughter is constantly reminding me about [my medications]. So, my daughter makes it easier for me to commit to my medications.”* [Participant 448]

When resources were limited, participants often borrowed blood pressure devices from their social circle:

“*[I do not have a blood pressure device], I use my wife's blood pressure monitor.”* [Participant 184]

“*[The blood pressure] device I have at home is my mother's, we both use it*.” [Participant 55]

Familial support for transportation and clinical interpretation was essential, enabling access to and understanding of healthcare:

“*I never go alone [to the clinic], my daughter is always with me, and she can translate.”* [Participant 450]

“*I do not have a car; my daughter picks me up [for my appointments*].” [Participant 155]

Instrumental support from social networks eased participants' adjustment to life in the US and removed barriers to hypertension management:

“*Yes, in the beginning [it was difficult getting used to life in America], however, my daughter is a great caregiver, and she is making my life easier in America.”* [Participant 448]

### Theme 3: the loss of emotional support and its impact on hypertension management

Participants overwhelmingly discussed how displacement, separation from loved ones, and loss of social networks had a detrimental impact on their emotional well-being and hypertension management. The sudden loss of support often led to feelings of isolation, depression, and worsening health.

“*[My refugee experience] affected me a lot. I left my country and people I know and love, and since then, the change caused my hypertension. Our lives have changed 180 degrees.”* [Participant 176]

“*Everything I have been through affected my hypertension. My hypertension started after I left Syria. I have lost a lot of loved ones and siblings, and my depression has caused my hypertension. For example, I haven't seen my siblings in almost 11 years. Whenever I get depressed and start crying, my blood pressure goes up.”* [Participant 4]

“*Yes, I strongly believe that the root cause of most illnesses in the world is mental health. Losing loved ones and a country and being scattered all over the world has a huge toll on our health as refugees. I have a daughter in Germany and 3 daughters and a son in Jordan. I have not seen them in 10 years.”* [Participant 150]

Participants compared the social life of their home countries to the isolation of the US, linking these changes to elevated stress and hypertension.

“*Social life is extremely limited in the US, unlike Iraq, where you can always find your neighbors helping you out and many friends and families. There is a life in the Middle East and a social ambiance that de-stresses us and makes us happy. Life is more serious in America; you come back from work at 6 pm, and then people are isolated and not socially active, as in Iraq, where you can find late-night outings and fun.”* [Participant 143]

However, rebuilding social relationships in the US helped alleviate feelings of isolation and improve their physical health:

“*[Social relationships] do affect my health. When I first arrived in the US, there weren't many Syrians. As a family, our social life was dead, and we barely knew anyone; it depressed us. After a while, there was an influx of Syrian refugees, and we ended up making many great relationships with Syrian families.”* [Participant 54]

“*[Things that might enhance my hypertension management include] distracting myself with activities I like to forget about my hypertension. Like, going out with friends. I noticed that my hypertension was exacerbated when the pandemic started, and I was stuck at home with anxiety... All these experiences have caused and exacerbated my hypertension.”* [Participant 6]

Empathy from HCPs in the clinical space also served as an important form of emotional support, reducing stress:

“*I like my doctor so much; she is so empathetic. Whenever we meet, I feel like we are friends, and she tries to deflate my worries and stress.”* [Participant 3]

## Discussion

This study examined how social connectedness and support influence hypertension management among Syrian and Iraqi refugees resettled in the US. Only one-third of participants reported feeling socially connected following resettlement, with lower-income refugees more often reporting such connections, despite prolonged stays in the US. Descriptively, higher annual household income appeared to co-occur with lower reported social connectedness in this sample; however, the mechanisms underlying this relationship remain unclear from the current data. Future research should explore whether this reflects differences in neighborhood composition, economic or work-related demands, or other structural factors among higher-income refugees, aligning with existing literature linking income and solitary behaviors (47, 48). Given the cultural and experiential diversity within refugee populations, future research should prioritize group-specific approaches and interventions, rather than treating refugees and other diverse populations as a monolith. The unequal distribution of Iraqi (*n* = 84) and Syrian (*n* = 23) participants in this study precluded meaningful group-level comparisons, and this remains an important direction for future investigation.

Social isolation, a well-documented significant risk factor for poor health (21, 22), emerged as a major concern within our study population and impacted hypertension management. Consistent with the U.S. Surgeon General's Advisory on the Healing Effects of Social Connection and Community (2023) (49), these findings highlight loneliness and isolation as public health concerns and determinants of community health and resilience. This is increasingly concerning for older adult refugees, who face compounded risks from social isolation, displacement, and chronic illness. Given the persistent income disparities among refugees in the US (50) and established links between inequality and poor population health (51), interventions must address economic and social barriers to hypertension management. Moreover, definitions of social connection should be culturally tailored, as perceptions of social connectedness vary by culture. Our findings suggest that in Arab culture, social connectedness often centers on collectivist identity and systems of belonging, as opposed to individualistic measures of social belonging, like individual friendships. This distinction is reflected in Table 1, where many participants who reported social disconnection still reported having close friends. Having individual friendships does not necessarily mean a participant feels a valued sense of belonging within a broader community. This nuance is especially important to recognize when measuring social connectedness in collectivist cultures.

Qualitative findings demonstrated that social networks strongly shape hypertension management. Informational support from HCPs and trusted social ties contributed to positive health management and understanding. This aligns with existing literature demonstrating that informational support reduces rates of uncontrolled hypertension (21) and can be conceptualized through the health belief model, a framework that informs numerous health behavior interventions. Furthermore, limited access to linguistically and culturally relevant health information was identified as a significant barrier, while culturally concordant resources and communication reduced perceived barriers to hypertension management (52). Participant perceptions of patient education from HCPs, as a means of adhering to prescribed regimens, underscored the significance of this approach. Participants also sought advice from medical professionals within their social networks, illustrating a unique form of in-group informational support characterized by familiarity and trust. Future investigations focusing on ways to integrate evidence-based treatment guidelines and informational support through existing support networks are needed. Additionally, Arab refugees exhibited a preference for HCPs who shared their gender, culture, or language, preferences that align with prior literature indicating that positive patient-provider communication improves medication adherence and clinical outcomes (53, 54). Empathetic care further fosters trust and adherence (53, 55, 56), validating patients' lived experience and promoting engagement (56–58). Refugees, who often carry the burden of displacement and trauma, may benefit greatly from receiving support from HCPs who provide empathetic, culturally responsive care (59).

Instrumental support, including transportation, assistance with blood pressure monitoring, and interpreter services, was a major facilitator of hypertension management. However, access to care and engagement with healthcare services were reliant on the level of support participants received. Findings suggest several avenues for future research on health and community intervention-related topics. Given participants' heavy reliance on family members for transportation, interpretation, and medication reminders, family-centered and empowerment-based care models warrant investigation in refugee populations. These care models, which involve professional interactions with caregivers to honor beliefs and cultural values, have demonstrated potential in improving health outcomes for non-refugee patients with hypertension (60, 61). Such models may be particularly relevant and warrant exploration among refugee patients, as instrumental support from social networks emerged as a key facilitator of hypertension management in this study. Shared medical appointments (SMAs), which enable families to attend joint medical appointments, have been shown to strengthen continuity of care and improve hypertension management among non-refugee patients (62). Considering this, further research assessing the feasibility of family-centered care models, including SMAs, may be warranted for refugee populations. Participants also identified transportation as a concrete barrier to care. Transportation support programs, such as transportation vouchers and complementary shuttle services, have been shown to reduce barriers to healthcare access in other chronic disease contexts, suggesting that transportation-based interventions should be explored for the refugee community (63).

Emotional support also emerged as a critical factor. Many participants attributed their hypertension to trauma, grief, and isolation resulting from family separation and displacement. Refugees experience disproportionately high rates of PTSD, depression, and anxiety (31), which can worsen chronic disease and psychosocial wellbeing, especially when exacerbated by social disconnection. Participants frequently described the emotional toll of family separation, which they attributed in part to difficulties with family reunification due to displacement and resettlement processes. These experiences of grief and isolation were perceived by participants as negatively affecting their emotional wellbeing and, by extension, their hypertension management. The relationship between specific immigration policies and clinical hypertension outcomes warrants direct investigation in future studies. The impact of such policies on health and hypertension management is further explored in a separate sub-study (64). Community-based interventions, which can serve as sources of emotional and social support, have been similarly associated with increased community connectedness, enhanced social support, improved stress-coping mechanisms, and decreased loneliness within refugee groups (65). Findings highlight the importance of future research on emotional support interventions, including group psychotherapy and expressive writing, which have demonstrated efficacy in improving physical and psychosocial illness in other populations (66).

To our knowledge, this is the first study to examine the role of social support in hypertension management among resettled Arab refugees in the US. Limitations include the small, localized sample and the use of a single survey item to assess perceived social connectedness alongside one validated scale, which may not comprehensively assess all forms of social support. As a result, the findings may not be generalizable to the broader refugee population and, thus, pose a potential bias in data analysis. Given the single-site sample and this study's focus on Arab cultural values, findings should be interpreted with caution and may not generalize beyond Arabic-speaking MENA refugee communities with comparable resettlement and post-resettlement refugee experiences. Descriptive patterns suggest that household income was the only sociodemographic characteristic that appeared to differ with social connectedness, though the limited sample size and descriptive nature of the data preclude any conclusions about this relationship. Considering this, future research should include larger, more socially diverse samples. Furthermore, self-reported data may be subject to social desirability bias, despite assurances of anonymity and confidentiality. Reflexivity is also acknowledged as a methodological consideration. Interviews were conducted in Arabic or English by trained bilingual researchers, including second-generation refugees with lived experience of forced migration, which likely facilitated trust and cultural concordance with participants. While this positionality strengthened rapport and may have reduced social desirability bias, researchers' shared cultural and linguistic backgrounds could also have introduced interpretive assumptions during thematic analysis. These potential influences were mitigated through independent coding, consensus-based codebook refinement, and iterative review by the full research team. Nonetheless, these findings establish a foundation for future research exploring various types of social support within this population.

## Conclusion

This study highlights the significant role of social support in hypertension management among Arab refugees, with participants describing its influence on adherence, access, and overall health outcomes. Findings emphasize that strengthening social connectedness, a key social determinant of health, should be a priority in refugee hypertension management. Displacement and isolation exacerbate barriers to care, underscoring the urgent need for sustainable, culturally tailored, community-based interventions that empower refugees to manage their hypertension while strengthening social connectedness. Community-centered programs can work to bridge informational, instrumental, and emotional support gaps and may contribute to improved health outcomes. In a greater context, findings highlight the importance of future research on emotional support interventions. Policy efforts to facilitate family reunification, integrate cultural humility and empathy into provider training, and address systemic barriers to care may support enhancement of refugee social connectedness, resilience, and health. Strengthening refugee social cohesion and connectedness, particularly through facilitating reunification with loved ones, may contribute to more successful assimilation and integration, chronic disease management, and long-term wellbeing among this population.

## Data Availability

The datasets presented in this article are not readily available because the data that support the findings of this study are not publicly available due to participant consent and data sensitivity. De-identified data may be made available upon reasonable request to the corresponding author, subject to review under applicable institutional and ethical guidelines. Requests to access the datasets should be directed to talrousan@health.ucsd.edu.

## Supplementary material

Supplementary Figure 1 Participant Flow Diagram for Quantitative Demographic Survey: STROBE Cross-Sectional Reporting Guideline Item 13.

Supplementary Figure 1 Note Of 756 individuals assessed for eligibility between April 2021 and April 2022, 130 met inclusion criteria. Twenty declined participation, leaving 110 enrolled. Three participants responded “I don't know” to the social connectedness question (Supplementary Appendix 1, Survey Question 10) and were excluded from analysis, yielding a final analytic sample of 107 participants.
