# Supplementary figures and images for: “I left my country and the people I love. It gave me hypertension”: a qualitative study of social support and hypertension management in refugees
Source: Front Public Health. 2026 Jul 15;14:1849878. doi: 10.3389/fpubh.2026.1849878 (PMC13416332; doi:10.3389/fpubh.2026.1849878)

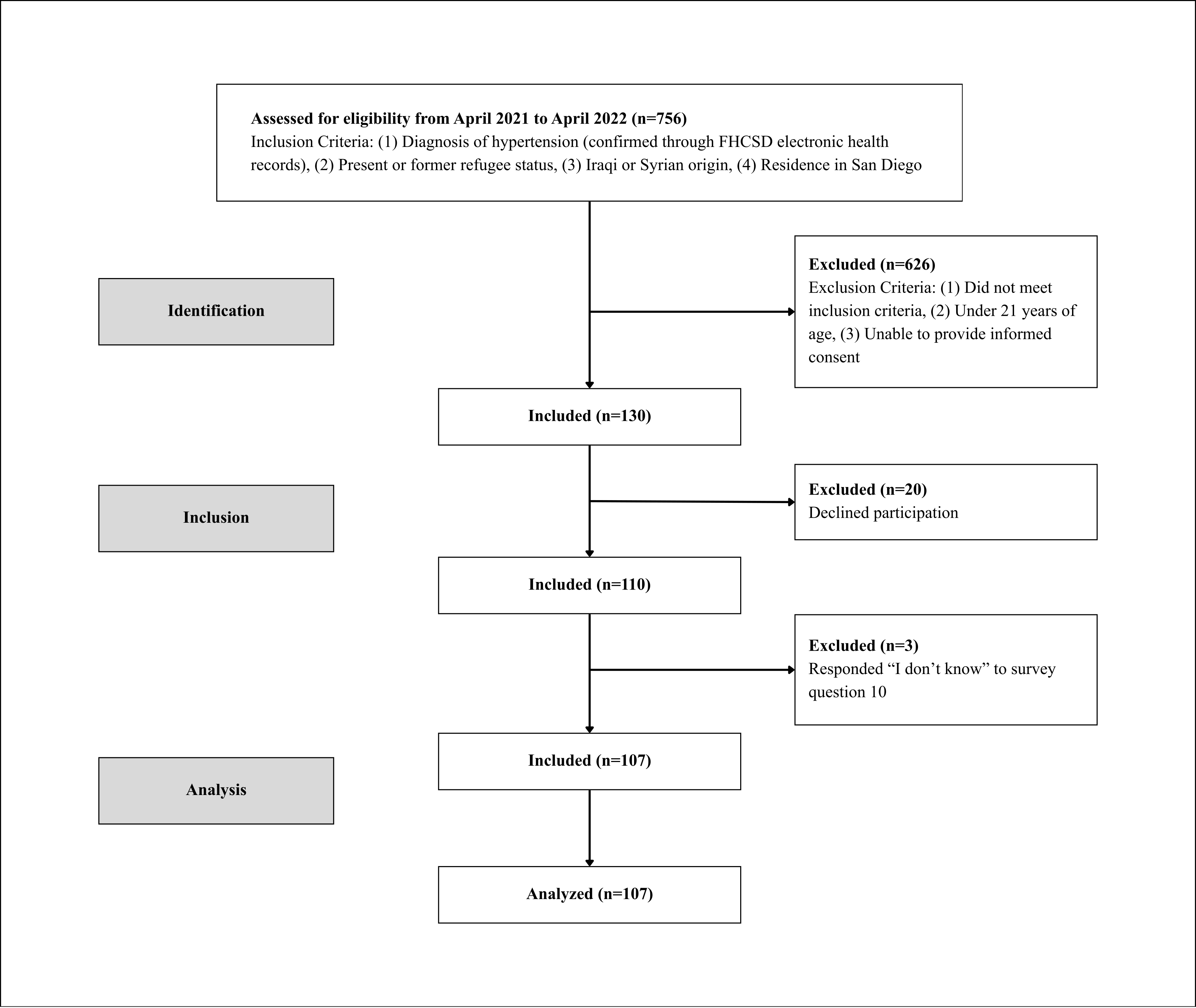

Supplement: Supplementary Figure 1 — Participant Flow Diagram for Quantitative Demographic Survey: STROBE Cross-Sectional Reporting Guideline Item 13. [file Image_1.tiff]
