# Supplementary material for: “I left my country and the people I love. It gave me hypertension”: a qualitative study of social support and hypertension management in refugees
Source: Front Public Health. 2026 Jul 15;14:1849878. doi: 10.3389/fpubh.2026.1849878 (PMC13416332; doi:10.3389/fpubh.2026.1849878)
Supplement: Supplementary Figure 1 Note — Of 756 individuals assessed for eligibility between April 2021 and April 2022, 130 met inclusion criteria. Twenty declined participation, leaving 110 enrolled. Three participants responded “I don't know” to the social connectedness question (Supplementary Appendix 1, Survey Question 10) and were excluded from analysis, yielding a final analytic sample of 107 participants. [file Table_1.docx]

Supplementary Material

# Supplementary Appendix 1

1. Date of birth
2. Gender?
   1. Male
   2. Female
   3. Other
3. Country of origin?
   1. Syria
   2. Iraq
4. Date of resettlement in the U.S.
5. Are you employed?
   1. Yes
   2. No
6. Annual household income?
   1. Less than $15,000
   2. $15,001-$25,000
   3. $25,001-$35,000
   4. $35,001-$50,000
   5. $50,000 or higher
7. What is the highest level of education you have attained?
   1. Less than high school
   2. High school
   3. Vocational certificate
   4. Bachelor's degree
   5. Post-graduate degree or higher
8. Are you able to read and write in Arabic?
   1. Yes
   2. No
9. Are you able to read and write in English?
   1. Yes
   2. No
10. Do you consider yourself socially connected to a specific community or group (e.g., the Muslim community network, refugee networks, home country networks, etc.)?
    1. Yes
    2. No
    3. I don’t know
11. If you think about your community, how many close friends do you have?
    1. 1
    2. 2
    3. 3
    4. 4
    5. 5 or more
12. In the case of an emergency, how many people can you turn to?
    1. 1
    2. 2
    3. 3
    4. 4
    5. 5 or more
13. What is the role of social support in your ability to manage hypertension? Did it get affected by your moving to the United States? How?
    1. Do you get help from others on how to use a home BP monitor? Take your medication?
    2. Do you use help from others in getting to or communicating with the doctor?
    3. Where do you get your information on hypertension from?
14. What are the difficulties in getting your blood pressure checked regularly?
    1. Probing questions:
       1. Do you have a blood pressure monitor at home?
       2. Are there any transportation issues with going to the doctor?
       3. Are there any language barriers at the clinic?
15. In what ways has the refugee experience impacted your hypertension management and your overall health, including social relationships?
    1. Probing questions:
       1. Was the move difficult to adjust to? Did that affect your hypertension?
       2. Are you still connected to your family back home?
