# Supplementary material for: “I left my country and the people I love. It gave me hypertension”: a qualitative study of social support and hypertension management in refugees
Source: Front Public Health. 2026 Jul 15;14:1849878. doi: 10.3389/fpubh.2026.1849878 (PMC13416332; doi:10.3389/fpubh.2026.1849878)
Supplement: Supplementary file 4 [file Table_3.docx]

STROBE Statement—Checklist of items that should be included in reports of ***cross-sectional studies***

|  | Item No | Recommendation | Page No |
| --- | --- | --- | --- |
| **Title and abstract** | 1 | (*a*) Indicate the study’s design with a commonly used term in the title or the abstract | Page 01 |
|  |  | (*b*) Provide in the abstract an informative and balanced summary of what was done and what was found | Page 01 |
| Introduction | | | |
| Background/rationale | 2 | Explain the scientific background and rationale for the investigation being reported | Page 02 |
| Objectives | 3 | State specific objectives, including any prespecified hypotheses | Page 02 |
| Methods | | | |
| Study design | 4 | Present key elements of study design early in the paper | Page 03, Page 05 |
| Setting | 5 | Describe the setting, locations, and relevant dates, including periods of recruitment, exposure, follow-up, and data collection | Page 03, Page 05 |
| Participants | 6 | (*a*) Give the eligibility criteria, and the sources and methods of selection of participants | Page 03, Page 05 |
| Variables | 7 | Clearly define all outcomes, exposures, predictors, potential confounders, and effect modifiers. Give diagnostic criteria, if applicable | Page 03 |
| Data sources/ measurement | 8* | For each variable of interest, give sources of data and details of methods of assessment (measurement). Describe comparability of assessment methods if there is more than one group | Page 03, Page 05 |
| Bias | 9 | Describe any efforts to address potential sources of bias | Page 03, Page 05 |
| Study size | 10 | Explain how the study size was arrived at | Page 03, Page 05 |
| Quantitative variables | 11 | Explain how quantitative variables were handled in the analyses. If applicable, describe which groupings were chosen and why | Page 4  Lines 136-158 |
| Statistical methods | 12 | (*a*) Describe all statistical methods, including those used to control for confounding | Page 03 |
|  |  | (*b*) Describe any methods used to examine subgroups and interactions | Not applicable |
|  |  | (*c*) Explain how missing data were addressed | Page 03 |
|  |  | (*d*) If applicable, describe analytical methods taking account of sampling strategy | Page 03 |
|  |  | (*e*) Describe any sensitivity analyses | Not applicable, descriptive statistics only |
| Results | | | |
| Participants | 13* | (a) Report numbers of individuals at each stage of study—eg numbers potentially eligible, examined for eligibility, confirmed eligible, included in the study, completing follow-up, and analysed | Page 03 |
|  |  | (b) Give reasons for non-participation at each stage | Page 03 |
|  |  | (c) Consider use of a flow diagram | Included as supplementary material (Supplementary Table 1) |
| Descriptive data | 14* | (a) Give characteristics of study participants (eg demographic, clinical, social) and information on exposures and potential confounders | Page 06 |
|  |  | (b) Indicate number of participants with missing data for each variable of interest | Page 05 (Table 1 Caption) |
| Outcome data | 15* | Report numbers of outcome events or summary measures | Page 06 |
| Main results | 16 | (*a*) Give unadjusted estimates and, if applicable, confounder-adjusted estimates and their precision (eg, 95% confidence interval). Make clear which confounders were adjusted for and why they were included | Not applicable, descriptive statistics only |
|  |  | (*b*) Report category boundaries when continuous variables were categorized | Page 03 |
|  |  | (*c*) If relevant, consider translating estimates of relative risk into absolute risk for a meaningful time period | Not applicable |
| Other analyses | 17 | Report other analyses done—eg analyses of subgroups and interactions, and sensitivity analyses | Not applicable |
| Discussion | | | |
| Key results | 18 | Summarise key results with reference to study objectives | Page 08, Page 09 |
| Limitations | 19 | Discuss limitations of the study, taking into account sources of potential bias or imprecision. Discuss both direction and magnitude of any potential bias | Page 09, Page 10 |
| Interpretation | 20 | Give a cautious overall interpretation of results considering objectives, limitations, multiplicity of analyses, results from similar studies, and other relevant evidence | Page 09, Page 10 |
| Generalisability | 21 | Discuss the generalisability (external validity) of the study results | Page 09, Page 10 |
| Other information | | | |
| Funding | 22 | Give the source of funding and the role of the funders for the present study and, if applicable, for the original study on which the present article is based | Page 09, Page 10, Page 11 |

*Give information separately for exposed and unexposed groups.

**Note:** An Explanation and Elaboration article discusses each checklist item and gives methodological background and published examples of transparent reporting. The STROBE checklist is best used in conjunction with this article (freely available on the Web sites of PLoS Medicine at http://www.plosmedicine.org/, Annals of Internal Medicine at http://www.annals.org/, and Epidemiology at http://www.epidem.com/). Information on the STROBE Initiative is available at www.strobe-statement.org.
